# Current challenges and practical aspects of molecular pathology for bone and soft tissue tumors

**DOI:** 10.1007/s00428-024-03736-5

**Published:** 2024-01-16

**Authors:** Enrique de Álava

**Affiliations:** 1 Institute of Biomedicine of Sevilla, IBiS/Virgen del Rocio University Hospital/CSIC/University of Sevilla/CIBERONC, 41013 Seville, Spain; 2 Department of Normal and Pathological Cytology and Histology, School of Medicine, University of Seville, 41009 Seville, Spain; 3 Department of Pathology, Virgen del Rocío University Hospital, Manuel Siurot S/N, 41013 Seville, Spain

**Keywords:** Molecular pathology, Sarcomas, Biomarkers, Diagnostics, Genomics

## Abstract

This review shows the extraordinary change molecular pathology has induced in the classification, diagnosis, and clinical practice of molecular pathologists dealing with sarcomas. We have primarily focused on the practical aspects of molecular studies and the current and mid-term challenges for our subspecialty, ending with ten tips for the next generation of sarcoma molecular pathologists.

## Introduction

### Brief description of molecular pathology and its significance in oncology

Mesenchymal neoplasms constitute a large group of tumor entities characterized by their taxonomic complexity and complex management. The publication of the 5th edition of the WHO classification of bone and soft tissue tumors in 2020 [1] and subsequent articles between 2020 and 2023 reflects the enormous progress in our knowledge of these tumors [2, 3]. The updating of taxonomic classifications, the redefinition of diagnostic criteria, the invention of new molecular diagnostic techniques, the development of prognostic indices, and the design of therapeutic targets correspond to the most critical innovations in translational research in the molecular pathology of mesenchymal neoplasms and, by extension, in the fight against cancer. Therefore, integrating molecular pathology in managing these tumors is a critical tool that links the development of scientific knowledge with diagnostic and therapeutic improvements in managing patients with mesenchymal neoplasms.

### Contextualization of soft tissue and bone tumors in molecular pathology

Molecular pathology has revolutionized our understanding of tumor biology to the point of exponentially increasing our knowledge of the natural history of neoplasms. Although each type of mesenchymal neoplasm usually presents several molecular alterations, some of them carry a greater weight in tumor biology. Basically, we can differentiate three groups of mesenchymal neoplasms based on their molecular pathology [3] (Table 1). The first group refers to the group of tumors that do not present a specific molecular alteration of clinical interest; i.e., there is no alteration sufficiently relevant for it to be considered of diagnostic and/or therapeutic importance (this is the case, for example, of undifferentiated pleomorphic sarcoma). In the second group, we would find neoplasms with recurrent molecular alterations, among which we can highlight gene fusions, point mutations, deletions, and amplifications (i.e., Ewing sarcoma). Finally, we would have a group of neoplasms with complex karyotypes. These can occur de novo or, more frequently, due to the degeneration of previously existing neoplasms with more favorable characteristics (i.e., malignant peripheral nerve sheath tumor). The presence of a complex karyotype is believed to originate in the loss of tumor suppressor genes of enormous importance, such as *RB1*, *NF1*, and *P53*, and in the phenomena of chromoanagenesis [4].

**Table 1:** Three main molecular types of bone and soft tissue sarcomas

Type of mesenchymal neoplasm	Molecular alterations	Examples
Tumors without specific molecular alterations	None	Undifferentiated pleomorphic sarcoma
Neoplasms with recurrent molecular changes	Gene fusions, mutations, deletions, amplifications	Ewing sarcoma
Neoplasms with complex karyotypes	Loss of tumor suppressor genes (e.g., RB, NF1, P53)	Malignant peripheral nerve sheath tumor

The mortality rate for high-grade metastatic sarcomas remains very high [5]. Sarcomas are highly heterogeneous morphologically, genetically, and in their behavior, so in addition to chemotherapy, which has a limited role in disease control, new strategies are needed for their treatment. In this sense, applying precision medicine strategies, which must start from a more precise diagnosis, is of extraordinary interest in such a heterogeneous group of tumors.

## Soft tissue tumors: practical molecular aspects

### The pivotal role of diagnostic biomarkers in molecular pathology of soft tissue tumors

Soft tissue tumors represent a diverse group of neoplasms with complex molecular underpinnings. Advances in molecular pathology have revolutionized our understanding and classification of these tumors, primarily due to the discovery and understanding of specific diagnostic biomarkers (see Table 2).

**Fig. 1:**
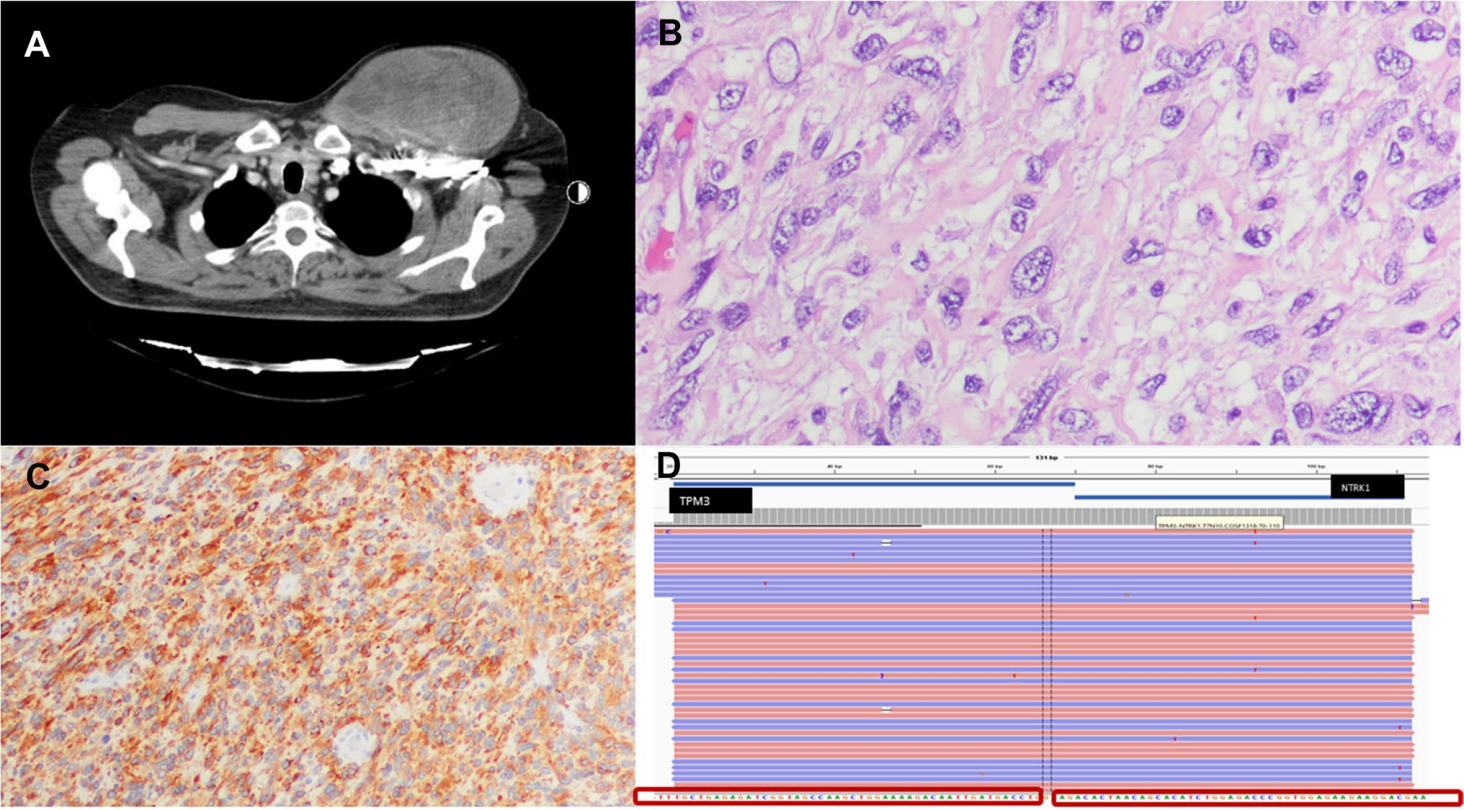
A 42-year-old man with an NTRK sarcoma. **A** Imaging shows a rapidly growing mass in the pectoral region. **B** It corresponds to a spindle/epithelioid cell sarcoma (H&E × 400). **C** Tumor cells show pan-NTRK expression (× 100). **D** Targeted sequencing shows *TPM3-NTRK1* fusions

- Lipogenic neoplasms [6]: Atypical pleomorphic and spindle-cell lipomatous tumors, previously variants of well-differentiated liposarcomas, are now distinctively identified by the absence of *MDM2* and *CDK4* gene amplification and, notably, the deletion of 13q14 and loss of the *RB1* gene in many cases.
- Fibroblastic and myofibroblastic neoplasms: The presence of the *NCOA2* gene rearrangement, leading to *AHRR::NCOA2* fusion in most angiofibroma of soft tissues [7], pinpoints its molecular pathology. Another critical discovery is the *EWSR1::SMAD3* fusion in *EWSR1*-positive fibroblastic tumors [8], providing clear diagnostic criteria.
- Fibrohistiocytic neoplasms: Molecular insights have led to a shift in classification. For instance, the once-termed malignant fibrous histiocytomas have been divided into multiple distinct entities.
- Vascular neoplasms: The identification of *GNAQ* or *GNA14* gene mutations in anastomosing hemangioma and the discovery of two main fusion types in epithelioid hemangioendothelioma, *WWTR1::CAMTA1* and *YAP1::TFE3* [9], have significantly advanced our diagnostic accuracy.
- Smooth muscle neoplasms: The expression of the viral EBER RNA in Epstein-Barr virus–positive smooth muscle tumors [10], leading to *MYC* overexpression, underlines its diagnostic significance. Additionally, in most cases, inflammatory leiomyosarcomas showcase a near-haploid karyotype, further refining our diagnostic approach.
- Striated muscle neoplasms: Newly identified fusions like *TFCP2* with *FUS* or *EWSR1* and *MEIS1::NCOA2* in rhabdomyosarcomas [11] have shifted our understanding of their origins and aggressiveness.
- Osteochondrogenic neoplasms: For instance, soft tissue chondromas are now known to harbor *FN1::FGFR* gene fusions in up to 50% of cases [12].
- Neoplasms of the nerve sheath: The malignant melanotic tumor of the nerve sheath is linked to Carney’s complex in a significant proportion of cases, underpinned by the loss of the tumor suppressor gene *PRKAR1A* [13].
- Other soft tissue neoplasms: The discovery of *NTRK* gene rearrangements (reviewed in 6) has been groundbreaking due to their therapeutic implications. The presence of these rearrangements is a pivotal diagnostic criterion for certain tumors (Fig. 1).
- Undifferentiated round cell sarcomas [14]: The emergence of advanced high-throughput methodologies has significantly reshaped our understanding and categorization of small round cell sarcomas (SRCSs). This evolution, fueled by the integration of extensive genetic, epigenetic, and transcriptomic insights along with progressive clinicopathological data and experimental frameworks, culminated in the establishment of a novel chapter dedicated to “undifferentiated SRCSs of bone and soft tissue” in the 2020 WHO classification for soft tissue and bone tumors. As these technologies evolve, they are expected to uncover even more uncommon SRCS variants.
- Predominantly, the most common fusion-driven entities that resemble Ewing sarcoma in morphology include round cell sarcomas characterized by the fusion of EWSR1 or FUS with non-ETS family genes (notably *EWSR1::NFATC2*, *FUS::NFATC2*, and *EWSR1::PATZ1*), sarcomas with CIC rearrangements (primarily *CIC::DUX4*), and sarcomas exhibiting BCOR genetic changes (chiefly *BCOR::CCNB3*). The consequences of these fusions on intracellular signaling pathways emphasize the shift in our understanding of tumor biology based on molecular findings [14].

**Table 2:** Soft tissue neoplasms and recently described genetic alterations

Neoplasm group	Tumor subtype	Genetic alterations
Lipogenic neoplasms	Atypical pleomorphic and spindle-cell lipomatous tumors	Absence of *MDM2* and *CDK4* gene amplification, deletion of 13q14 and loss of RB1 gene in many cases
Fibrogenic and myofibrogenic neoplasms	Most angiofibroma of soft tissues, *EWSR1*-positive fibroblastic tumors	*NCOA2* gene rearrangement, leading to *AHRR::NCOA2* fusion; *EWSR1::SMAD3* fusion; sarcomas with KMT2A gene rearrangements; *PRRX1::NCOA1* fusion
Fibrohistiocytic neoplasms	Soft tissue giant cell tumors, malignant fibrous histiocytomas	Distinction based on absence of H3-3/H3F3 gene mutations
Vascular neoplasms	Anastomosing hemangioma, epithelioid hemangioendothelioma	*GNAQ* or *GNA14* gene mutations; *WWTR1::CAMTA1* and *YAP1::TFE3* fusions
Smooth muscle neoplasms	Epstein-Barr virus-positive smooth muscle tumors, inflammatory leiomyosarcomas	Viral EBER RNA expression in Epstein-Barr virus-positive tumors; near-haploid karyotype in inflammatory leiomyosarcomas
Striated muscle neoplasms	Rhabdomyosarcomas, inflammatory rhabdomyoblastic tumor	*TFCP2* with *FUS* or *EWSR1* and *MEIS1::NCOA2* fusions in rhabdomyosarcomas
Osteochondrogenic neoplasms	Soft tissue chondromas	*FN1::FGFR* gene fusions in up to 50% of cases
Neoplasms of the nerve sheath	Malignant melanotic tumor of the nerve sheath	Linked to Carney’s complex, loss of *PRKAR1A* gene
Other soft tissue neoplasms	NTRK sarcoma	*NTRK* gene rearrangements with therapeutic implications
	GLI-1 altered mesenchymal neoplasms	GLI-1 rearrangements or amplifications
Undifferentiated round cell sarcomas	Ewing sarcoma	*FET::ETS* fusions
	EWSR1::non-ETS sarcomas	*EWSR1::NFATC2, EWSR1::PATZ1*
	CIC-rearranged sarcomas	*CIC::DUX4*
	Sarcomas with BCOR alterations	*BCOR::CCNB3*

After the 5th edition of the WHO classification (2020–2023), the realm of soft tissue tumor molecular pathology witnessed further revelations:

- Fibrogenic neoplasms: Sarcomas with *KMT2A* gene rearrangements and fibroblastic tumors with *PRRX1::NCOA1* fusion have enriched the spectrum of soft tissue tumors [15].
- Fibrohistiocytic neoplasms: The distinction of soft tissue giant cell tumors from their bony counterparts based on the absence of *H3-3/H3F3* gene mutations is another testament to the precision offered by molecular biomarkers [16].
- Striated muscle neoplasms: The inflammatory rhabdomyoblastic tumor, a newly recognized entity, embodies the influence of molecular pathology in refining our understanding of soft tissue tumor classification [17].
- Other soft tissue neoplasms: The identification of *EWSR1::SSX2* fusion in a subset of undifferentiated soft tissue sarcomas [18], *NUTM1* gene rearrangements in colorectal sarcomas, and *FN1* gene rearrangements in chondroid neoplasms underscores the relentless evolution of soft tissue tumor classification. Glioma-associated oncogene 1 (*GLI1*), a transcription factor activated by the Sonic hedgehog pathway, plays a role in the development of various tumors, including gliomas, alveolar rhabdomyosarcomas, and osteosarcomas. *GLI1* amplifications and gene fusions are also found in diverse mesenchymal tumors like pericytoma with t(7;12), gastroblastoma, plexiform fibromyxoma, and a new category of GLI1-altered mesenchymal neoplasms. This group includes “nested glomoid neoplasm,” a new tumor type with unique architecture, and a range of low to high-grade neoplasms, some resembling myoepithelial carcinoma. Pericytomas with t(7;12) and nested glomoid neoplasms have distinct morphologies and immunohistochemical profiles, expressing markers like S100, SMA, CDK4, and MDM2. GLI1 immunohistochemistry can aid in diagnosing these rare tumors, potentially eliminating the need for molecular testing [19].

Integrating diagnostic biomarkers into the classification of soft tissue tumors has transformed the landscape of tumor diagnosis, prognosis, and treatment. It underscores the importance of a multidisciplinary approach, combining histopathology with molecular pathology, for the optimal management of patients with these neoplasms.

## Bone tumors: practical molecular aspects

Mesenchymal bone neoplasms are a group of tumors originating from the bone’s mesenchymal tissue. These neoplasms vary in their aggressiveness, clinical presentation, histology, and genetics. Below is a comprehensive review of the different categories of these neoplasms and their associated molecular characteristics (see also Table 3):

1. Chondrogenic neoplasms
  *Chondromyxoid fibroma*: This neoplasm is linked to rearrangements of the *GRM1* gene. Overexpression of *GRM1* indicates the diagnosis of chondromyxoid fibromas [20]. However, a small percentage does not show this overexpression, suggesting the possibility of other genetic alterations.
    *Synovial chondromatosis*: associated with the *FN1::ACVR2A* and *ACVR2A::FN1* fusions [20]. These fusions are present in most benign synovial chondromatosis and some malignant cases.
2. Osteogenic neoplasms
  *Osteoid osteoma*: Characterized by the presence of *FOS* rearrangement in most cases [21]. A common neoplastic spectrum with osteoblastoma and epithelioid hemangioma is postulated. The *FOS* family plays a crucial role in cellular transcription. Although the diagnosis of almost all osteoid osteomas does not require a demonstration of FOS rearrangements, their detection can be useful in selected cases in which a clear radiology-pathology correlation is missing.
    *Osteoblastoma*: Similar to osteoid osteoma, it shows rearrangement of *FOS*. Also observed, albeit less frequently is the rearrangement of *FOSB* [21].
3. Giant cell–rich neoplasms
  *Non-ossifying fibroma*: A subset of these tumors originates due to neurofibromatosis type 1 and Jaffe-Campanacci syndrome. They are associated with mutations in the *NF1* and *KRAS* genes. Additionally, they are characterized by mutations in *KRAS* and *FGFR1* [22].
4. Notochordal neoplasms
  *Poorly differentiated chordoma*: This neoplasm shows a homozygous *SMARCB1/INI1* gene deletion. A small fraction of cases show gene loss without detectable mutation [23]. Furthermore, some cases have a codeletion of the *EWSR1* gene.
5. Other bone neoplasms
  *Adamantinoma*: This neoplasm displays both numerical and structural chromosomopathies. They are associated with trisomies and chromosomal translocations. The dedifferentiation process in these neoplasms is linked to the loss of *P53* and the acquisition of a complex karyotype.

**Table 3:** Bone neoplasms and recently described genetic alterations

Neoplasm group	Tumor subtype	Genetic alterations
Chondrogenic neoplasms	Chondromyxoid fibroma	Linked to rearrangements of the GRM1 gene
	Synovial chondromatosis	Associated with FN1::ACVR2A and ACVR2A::FN1 fusions
Osteogenic neoplasms	Osteoid osteoma	FOS rearrangement in most cases
	Osteoblastoma	Shows rearrangement of FOS and less frequently FOSB
Giant cell–rich neoplasms	Non-ossifying fibroma	Mutations in KRAS and FGFR1
Notochordal neoplasms	Poorly differentiated chordoma	Homozygous SMARCB1/INI1 gene deletion
Other bone neoplasms	Adamantinoma	Displays numerical and structural chromosomopathies

Identifying and understanding these neoplasms’ genetic and molecular alterations provide a deeper insight into their biology and potential therapeutic interventions.

## Molecular diagnostic techniques in clinical practice of bone and soft tissue pathology

Molecular diagnostics have markedly enhanced our ability to classify and diagnose neoplastic entities, particularly with the refinement of tumor taxonomy. This progress is exemplified by the GENSARC study [24], which indicated that molecular investigations altered initial diagnostic conclusions in about 14% of sarcoma cases made by specialized pathologists. Let us delve deeper into the panorama of molecular diagnostic techniques, both traditional and avant-garde.

### Traditional molecular techniques (reviewed in [25])

**Fig. 2:**
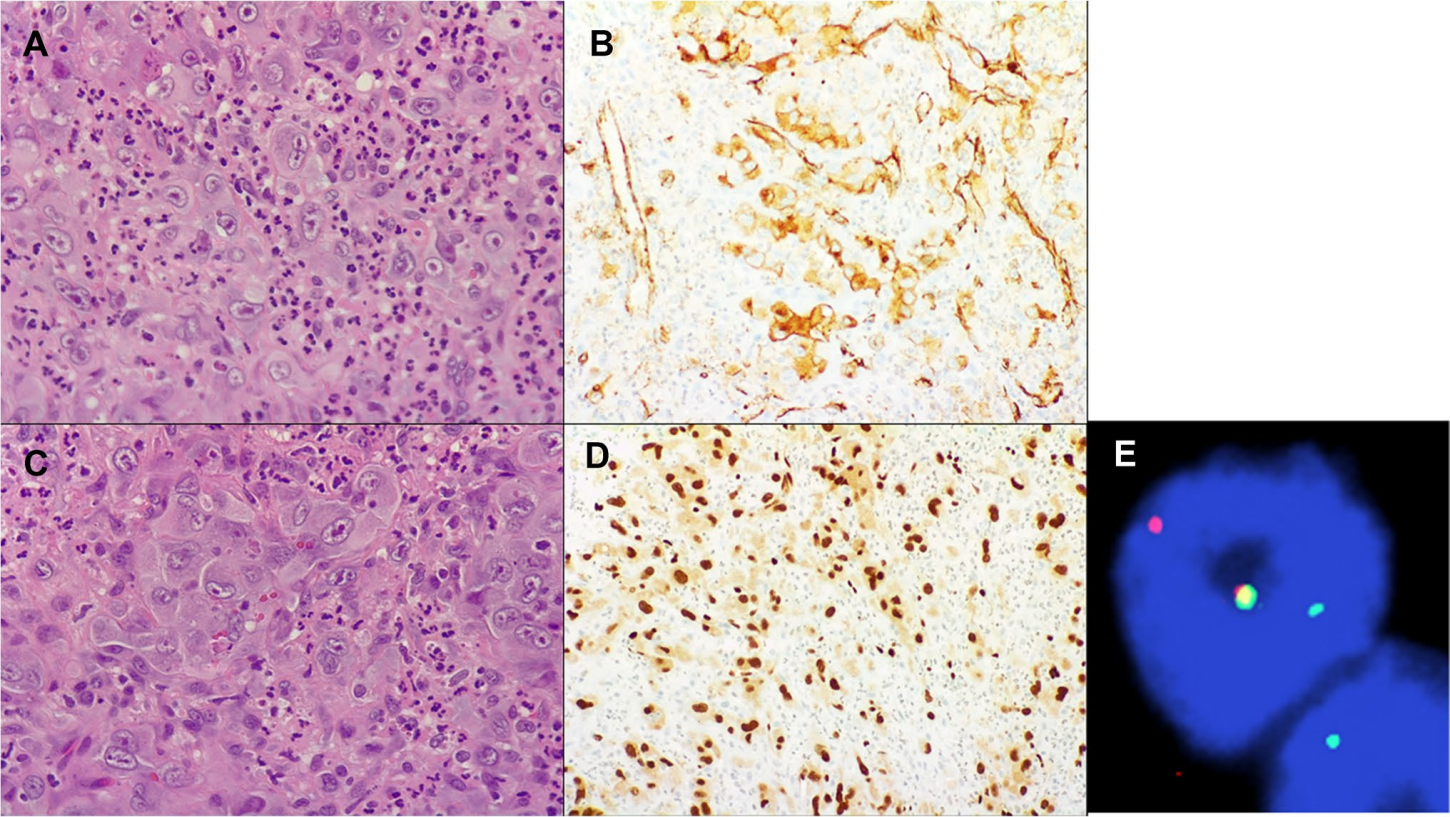
A 70-year-old male with mass in the femur and soft tissues and lesions in the lung and spleen. **A** Epithelioid cells with prominent nucleoli (H&E × 100). **B** CD31 expression (× 100). **C** Cytoplasmic lumina with red blood cells (H&E × 100). **D** ERG expression (× 100). **E** FISH analysis shows *WWTR1* rearrangements, confirming the diagnosis of epithelioid hemangioendothelioma

**Fig. 3:**
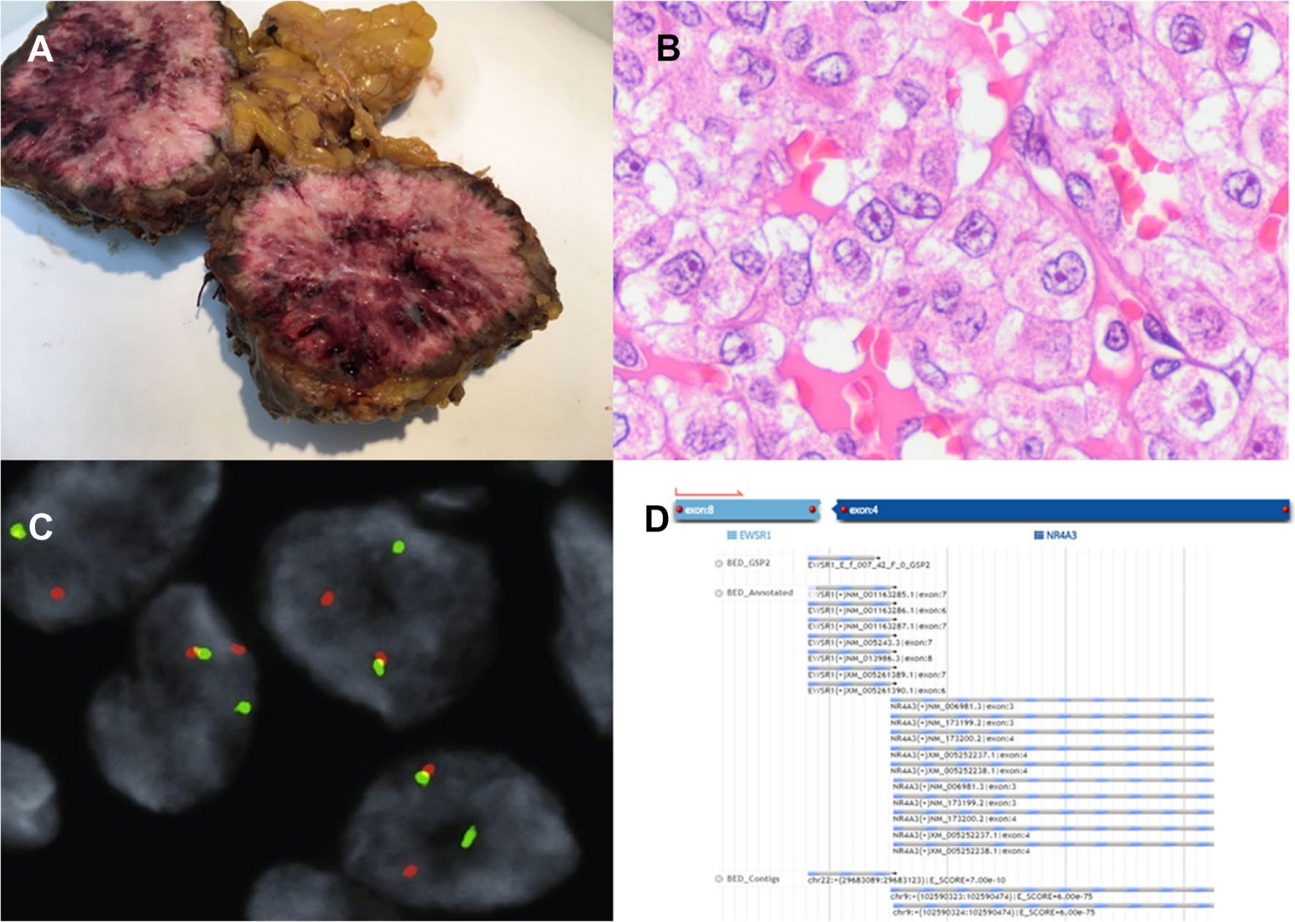
A 75-year-old man with a high-grade extraskeletal myxoid chondrosarcoma. **A** A 6-cm intramuscular mass in the abdominal wall. **B** Epithelioid high-grade neoplasm (H&E × 400). **C** FISH analysis showing *NR4A3* rearrangements. **D** *EWSR1::NR4A3* fusions were detected by targeted sequencing

- Karyotyping: A foundational technique, karyotyping was paramount in identifying the earliest chromosomal deviations in sarcomas. Nevertheless, its resolution, limited to around five megabases, impedes the recognition of specific gene mutations, relegating it to a more ancillary role in contemporary diagnostics.
- Comparative genomic hybridization (CGH): This technique can spotlight DNA amplifications and deletions. However, its limited prowess in pinpointing point mutations and gene fusions has confined its use to specific cases and research contexts.
- Fluorescence in situ hybridization (FISH): Widely employed for detecting translocations and amplifications, FISH, in tandem with PCR, stands as a cornerstone in the current diagnostic landscape (Fig. 2). Yet, its blind spots include an inability to discern discrete gene mutations and some proximate gene fusions [14] (Fig. 3).
- Polymerase chain reaction (PCR): A linchpin in many diagnostic labs, PCR has served as the molecular technique of choice for routine diagnostic procedures. However, it requires a prior understanding of the mutation under scrutiny and has constraints in detecting alternate partner genes.

### Modern molecular techniques

**Fig. 4:**
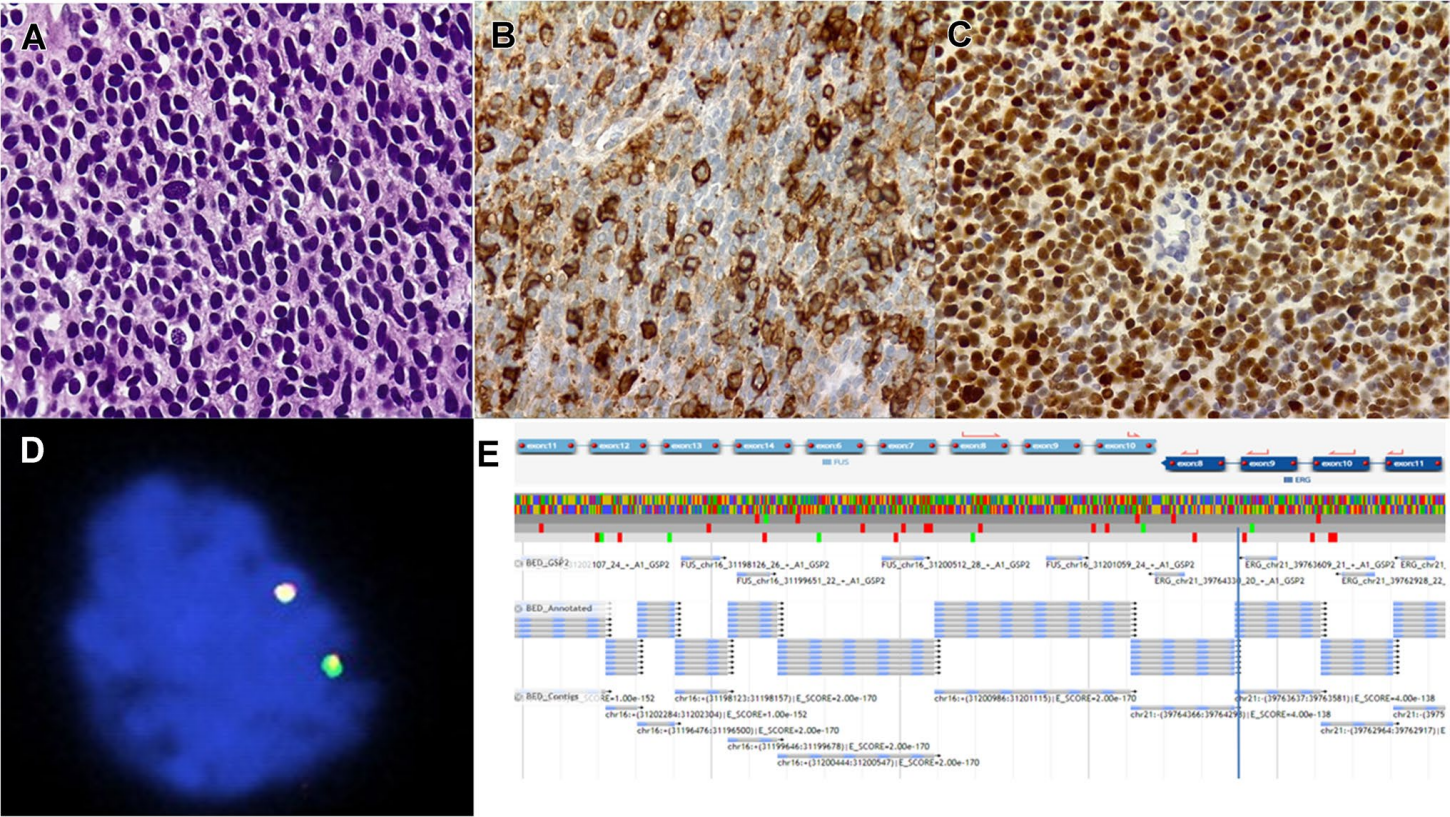
A 19-year-old woman with a vertebral mass. **A** Small cell sarcoma with round to oval cells. **B** Patchy CD99 staining (× 100). **C** Intense and diffuse expression of NKX2.2 (× 100). **D** FISH analysis with an *EWSR1* breakapart probe did not show *EWSR1* gene rearrangements. **E** Targeted sequencing, however, showed *FUS::ERG* fusions, confirming Ewing sarcoma diagnosis and showing a common pitfall of *EWSR1* breakapart FISH probes in *ERG*-rearranged Ewing sarcomas

- Next-generation sequencing (NGS): A quantum leap in genetic analysis, NGS allows for the parallel sequencing of vast DNA fragments. Its precision is further honed with targeted versions such as “gene panels by hybridization and capture” and “massive amplicon sequencing,” both of which can accurately identify alternate fusion partner genes (Fig. 4) [26].
- NanoString: A paradigm shift, this method quantifies RNA directly, bypassing the need for retrotranscription or prior amplification, using DNA probes adorned with fluorescent barcodes [27]. Especially adept at analyzing subpar RNA samples, it parallels mass sequencing in identifying a diverse array of partner gene fusions.
- Non-targeted massive sequencing: Offering an expansive purview, these techniques can decode any genetic rearrangement without prior knowledge. This category encompasses the holistic RNA-Seq, WES, and WGS techniques. The “nanopore” method is a groundbreaking offshoot, which deduces mutations by discerning ionic current shifts as DNA fragments traverse a nanoporous protein membrane.
- Methylome studies: These studies probe methylation patterns beyond mere genetic material, offering a more resilient and granular analysis [28]. Their binary approach, focusing on methylation or its absence, furnishes a unique lens for diagnostics.

### Recommendation for molecular testing in bone and soft tissue sarcomas

**Fig. 5:**
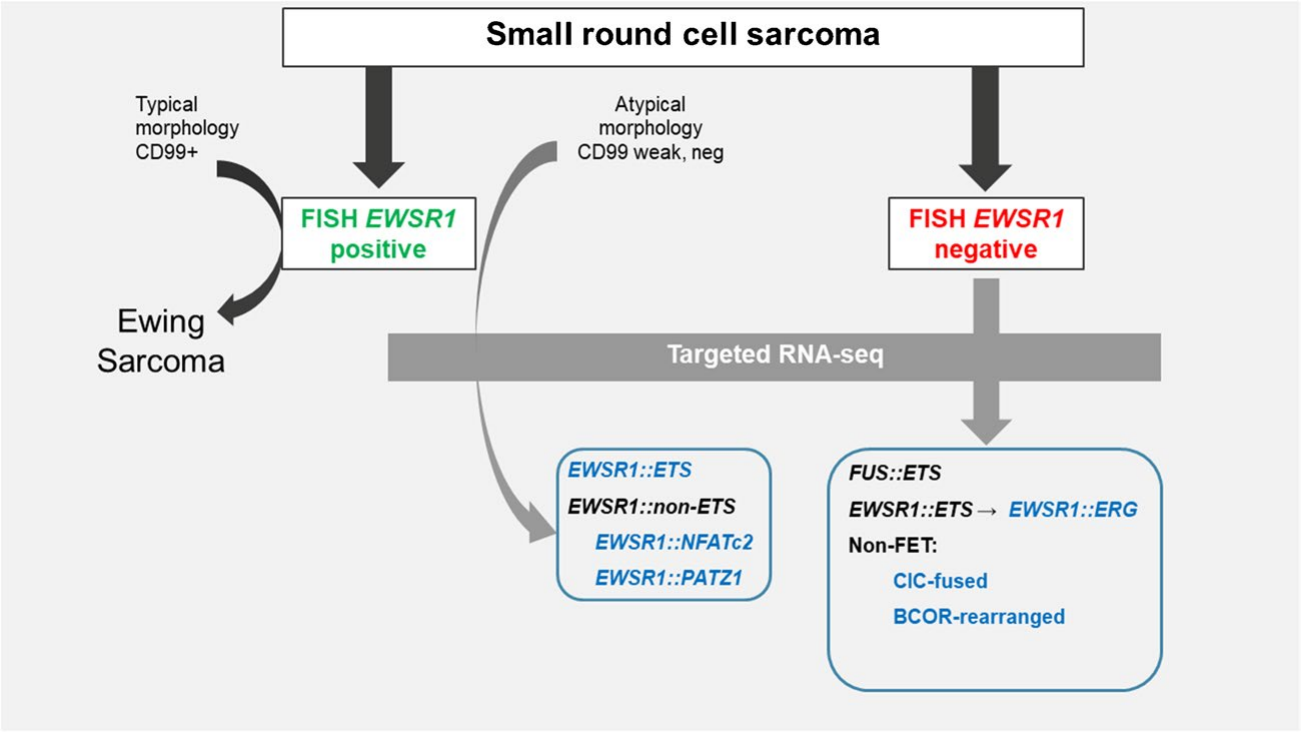
Diagnostic algoritm of small round cell sarcomas in our instituton integrates immunohistochemistry, FISH, and NGS assays to achieve a precise diagnosis

- Consider any molecular result in the right clinical and pathological context. Although some gene fusions are very specific to a particular tumor type (e.g. *EWSR1::FLI1* in Ewing sarcoma), other gene fusions are much more non-specific, such as *ETV6::NTRK3*, which can be seen not only in sarcomas but also in leukemias or carcinomas. Molecular findings should, therefore, never be evaluated in isolation, but always in the appropriate clinical and morphological context.
- Perform a foundational assessment with traditional techniques.
  - Begin with FISH, which is widely used for detecting translocations and amplifications, especially when specific genetic alterations or translocations are suspected.
  - Use PCR where a particular mutation is suspected, given its widespread use and the requirement of prior knowledge of the mutation.
- Make a judicious use of advanced analysis with modern techniques.
  - Next-generation sequencing (NGS) should be considered, primarily with a targeted approach. These allow for a comprehensive genetic analysis, are adept at identifying alternative fusion partner genes, and could be invaluable when a broader genetic landscape needs examination.
  - In cases with low-quality RNA samples or when a more expansive genetic view is needed, NanoString is recommended due to its ability to quantify RNA and identify diverse partner gene fusions directly.
  - For sarcomas of uncertain classification or when a holistic view of the genetic material is required, non-targeted massive sequencing techniques like RNA-Seq, WES, and WGS can be employed. The “nanopore” method [29], while more avant-garde, can offer a unique perspective by deducing mutations from ionic current shifts.
- Have supplemental analysis available: Methylome studies can be considered an auxiliary diagnostic tool, especially when the genetic material is compromised, providing a robust and detailed analysis based on methylation patterns.
- Always have a holistic or integrative consideration: Despite the advancements in molecular diagnostics, the foundation of sarcoma diagnosis remains rooted in histopathological findings. Molecular pathology should be used as a complementary tool, enhancing the specificity and accuracy of the diagnosis (Fig. 5).
- Be aware of cost and infrastructure: While modern techniques might seem resource-intensive, their potential efficiency, especially in complex sarcoma cases, could render them more cost-effective in the long run. When selecting a testing strategy, balancing the cost, available infrastructure, and diagnostic precision are essential.
- Immunohistochemistry can constitute an excellent surrogate of molecular genetics. Over the last decade, advancements in molecular genetics have revolutionized diagnostic approaches, leading to the development of novel, cost-effective, and rapid diagnostic tests using immunohistochemical stains. These new immunohistochemical markers are broadly classified into three categories: proteins indicative of genetic alterations such as PDGFRA, SMARCB1 [INI1], H3K27me3, SMARCA4 [BRG1], β-catenin, MDM2, MYC, RB1, CDK4, and SDHB; protein products resulting from gene fusions including STAT6, TFE3, ALK, FOSB, BCOR, DDIT3, SS18::SSX, CAMTA1, CCNB3, and pan-TRK; and diagnostic markers identified by gene expression profiling, such as MUC4, DOG1, NKX2-2, TLE1, SATB2, and ETV4. These advancements have significantly enhanced the speed and precision of diagnostics, particularly in the realm of sarcoma identification and classification [2, 3].

In summary, a layered approach, integrating traditional and modern techniques, can provide a comprehensive and accurate molecular diagnosis for bone and soft tissue sarcomas.

### Reporting

The results of ancillary tests (e.g., immunohistochemistry (IHC) or molecular evaluations) should be included in the report where relevant. This is the case, for example, for the detection of translocations in round cell sarcomas, isocitrate dehydrogenase (*IDH1* and *IDH2*) mutations in conventional chondrosarcoma, and *MDM2* amplification in low-grade intramedullary and parosteal osteosarcoma. The International Collaboration for Cancer Reporting [30] provides guidelines for standardized pathology reporting of soft tissue sarcomas [31]. It reminds us that molecular test results should be integrated into biopsy or resection reports of osteoarticular tumor pathology.

## Current challenges faced by molecular pathologists in bone and soft tissue Pathology(BSTPath) and significant concerns

### Managing discrepancies

In cases where sarcoma diagnosis reveals discrepancies between results from different molecular techniques (like FISH vs NGS) or between molecular and histological/immunohistochemical findings, a multifaceted and cautious approach is recommended:

1. Multidisciplinary review: Engage a multidisciplinary team including pathologists, molecular biologists, radiologists, and oncologists. This team can provide diverse perspectives and expertise, comprehensively analyzing all findings.
2. Re-evaluate clinical and radiological data: Reassess the patient’s clinical history and radiological data. Sometimes, additional clinical context or imaging studies can provide insights that help reconcile conflicting results.
3. Repeat or confirm tests: If feasible, repeat the tests that show discrepancies. For instance, if there is a mismatch between FISH and NGS results, consider repeating these tests or employing additional methodologies for confirmation.
4. Integrate histological and molecular data: The histological context can sometimes provide essential insights that guide the interpretation of molecular results. Ensure that molecular findings are correlated with histological and immunohistochemical features.
5. Consider technical limitations: Understand the limitations of each technique. For instance, FISH is highly specific but may miss broader genomic alterations that NGS can detect. Conversely, NGS is comprehensive but may miss focal alterations detectable by FISH.
6. Consultation with external experts: In particularly challenging cases, seeking a second opinion from external experts or reference laboratories can be invaluable.
7. Patient monitoring and follow-up: In cases of unresolved discrepancies, close monitoring of the patient with frequent follow-ups may be necessary. This approach can help detect any progression or changes that might clarify the diagnosis.
8. Document and report findings: Careful documentation of all findings and the decision-making process is crucial. This can be valuable for future reference, especially if the patient’s clinical situation evolves.
9. Continued research and learning: Stay updated with the latest research and advancements in sarcoma diagnostics. New discoveries and technologies might provide solutions to current diagnostic challenges.

### Setup of the pre-analytical phase in sarcomas

Sarcomas, especially those arising from bone tissues, present unique challenges during the biopsy processing phase. Bone sarcomas, in particular, often require decalcification processes to prepare the tissue for histological examination. However, decalcification can adversely affect the quality of nucleic acids, complicating subsequent molecular analysis [32]. This makes the choice of decalcification agent and duration of the process pivotal. In addition to this, the intrinsic nature of sarcomas being deep-seated tumors further complicates biopsy collection. Proper handling becomes paramount, given the diverse subtypes of sarcomas, each with distinct molecular profiles. Preserving RNA integrity in these samples is essential, especially when gene fusion detection, a hallmark of many sarcoma subtypes, is anticipated. As such, the pre-analytical phase requires careful orchestration of multiple steps, ensuring the best possible preservation of molecular details.

### Ensuring access to the tests of choice for sarcomas

With over 50 diverse subtypes, sarcomas present a tapestry of unique genetic alterations. While choosing the proper test is vital (see the “Recommendation for molecular testing in bone and soft tissue sarcomas” section), ensuring that these advanced diagnostic tools are equitably available to the general population becomes equally crucial. Whether it is the specificity of FISH for detecting specific translocations or the comprehensive capability of NGS to survey the broader genomic landscape, the real challenge lies in having access to these tests. Healthcare systems and policies must prioritize the widespread availability of these sophisticated diagnostics. This equitable distribution ensures that every patient, regardless of socio-economic status or geographical location, has a fighting chance at accurate diagnosis and targeted therapy. Moreover, a keen understanding of sarcoma histopathology and its potential molecular underpinnings underlines the importance of continuous training and updates for pathologists and technicians involved in sarcoma diagnostics.

### Optimizing the management of sarcoma samples

Given the heterogeneity of sarcomas, it is essential to obtain representative tissue samples. Ensuring that molecular tests do not exhaust these samples, especially when repeated biopsies are not feasible, is paramount. Multigene tests, as opposed to unigene tests, ensure that the original paraffin block of the diagnostic biopsy is not exhausted by repetitively accessing it each time a single-gene test is needed.

### Circulating biomarkers in sarcomas

The emerging field of liquid biopsies [33], which includes the analysis of circulating biomarkers such as cell-free DNA (cfDNA), circulating tumor DNA (ctDNA), circulating tumor cells (CTCs), and specific proteins, holds significant promise for sarcomas [34]. These non-invasive tests, derived primarily from blood samples, can potentially provide invaluable insights into the molecular landscape of a sarcoma without the need for a traditional tissue biopsy. For sarcomas, these liquid biopsies could aid in early diagnosis, monitoring treatment responses, and detecting recurrences. They might even unveil potential therapeutic targets or resistance mechanisms in real time. However, the inherent rarity and heterogeneity of sarcomas pose distinct challenges. Given the myriad subtypes of sarcomas with unique genetic and molecular characteristics, standardizing and validating liquid biopsy protocols become a complex endeavor. Furthermore, due to the deep-seated nature of many sarcomas, the amount of ctDNA shed into the bloodstream might be lower than in more prevalent cancers, which can affect the sensitivity of these tests. Therefore, while liquid biopsies present a revolutionary avenue for sarcoma diagnostics and management, comprehensive research and methodological advancements are needed to realize their full potential.

### Immunotherapies and sarcomas

While immunotherapies show promise in many cancers, their role in sarcomas is still evolving (reviewed in 34). Molecular pathologists play a pivotal role in researching the landscape of sarcomas, primarily in identifying and validating biomarkers that can guide immunotherapy. Through advanced techniques, they are adept at characterizing the prevalence of immune cells and discerning expression patterns of immune checkpoints like PD-1/PD-L1. These biomarkers, once validated, can be instrumental in determining the most suitable therapeutic strategies. However, given the complexity and heterogeneity of sarcomas, molecular pathology must continue its exploration and validation of new biomarkers to refine further and personalize immunotherapeutic interventions in these patients.

### Novel issues in sarcoma treatment

As targeted therapies for sarcomas emerge, understanding the molecular drivers, resistance mechanisms, and potential combination strategies becomes essential for molecular pathologists. For instance, sequencing of receptor tyrosine kinases (RTKs) like *KIT* and *PDGFRA* in gastrointestinal stromal tumors (GISTs) can guide the use of targeted therapies like imatinib. However, as tumors might acquire resistance to these therapies, pathologists play a critical role in detecting secondary mutations that could necessitate a switch in treatment strategy. This deep molecular insight ensures precise initial treatment selection and dynamic therapy adjustments based on the tumor’s evolving molecular profile, optimizing patient outcomes. These observations underscore the necessity of tailored tumor profiling for each patient to pinpoint active signaling pathways, moving beyond blanket treatment approaches toward individualized, versatile treatment plans [35]. Trials that match a specific therapy to shared oncogenic drivers across different diseases, like the CREATE trial, reflect this personalized approach [36]. Furthermore, understanding patient-to-patient differences in drug metabolism and response can be instrumental in anticipating and counteracting resistance mechanisms [37].

### Multidisciplinary tumor boards for sarcomas

Due to their intricate nature and myriad subtypes, sarcomas necessitate a collaborative approach to decision-making processes. Central to this collaboration is the multidisciplinary tumor board, where diverse specialists come together to discuss and design the optimal treatment plan for patients. Molecular pathologists play a pivotal role in these boards, as their detailed molecular insights can dictate the direction of treatment [38]. For instance, if a molecular pathologist identifies a specific genetic mutation that makes a particular sarcoma subtype responsive to a targeted therapy, this information must be communicated in an accessible and understandable manner. Radiologists, for example, might need to understand the potential growth patterns or metastatic tendencies associated with that mutation. At the same time, surgical oncologists might adjust their strategies based on the predicted aggressiveness or behavior of the tumor. Additionally, medical oncologists can tailor their chemotherapeutic regimens based on these insights. Thus, effective communication within the board ensures that the patient receives a holistic, informed, and precise treatment strategy, maximizing therapeutic success and potentially improving outcomes.

### Setting up national NGS networks for sarcomas

Creating a national framework for the molecular diagnostics of sarcomas is no small task, given the heterogeneity and intricacy of these tumors. Such networks provide standardized diagnostic protocols and ensure that even the less common sarcoma subtypes receive the attention they deserve. A stellar example of this approach’s success is seen in the efforts of the French sarcoma group, which has achieved remarkable progress in diagnosis and therapeutic strategies for sarcoma patients through their consolidated efforts. Similarly, Spain is making significant strides with projects such as IMPERAS (*Estudio del IMPacto En supervivencia y calidad de vida de la Revisión centralizada del diagnóstico Anatomopatológico en Sarcomas de partes blandas*; Study of the Impact on Survival and Quality of Life of Centralized Review of Pathologic Diagnosis in Soft Tissue Sarcomas) [39], aiming to streamline sarcoma diagnostics and research. This endeavor has gained momentum, especially with the additional support from AECC (Spanish Association Against Cancer), extending its reach and impact. These national initiatives underscore the importance of collaborative and standardized molecular diagnostic efforts in improving sarcoma patient outcomes. By leveraging the latest molecular insights and ensuring their widespread accessibility, these networks are pivotal in advancing sarcoma care nationally.

In conclusion, the challenges in molecular pathology take on added intricacy in the realm of sarcomas due to their diversity and complexity. Addressing these issues requires a concerted effort, a deep understanding of sarcoma biology, and a commitment to continuous learning in this rapidly evolving field.

## Short and mid-term perspectives

### Comprehensive genome profiling

The limited availability of effective targeted treatments for most types of sarcomas can, in part, be addressed by expanding our knowledge of the genetic mutations found in mesenchymal tumors [29, 40]. These tumors have not been as extensively studied as those originating from epithelial and neural tissues. Up until now, genetic research in sarcomas, including projects like The Cancer Genome Atlas (TCGA), has been constrained by small sample sizes, a focus on early-stage disease, a narrow range of histologies (like liposarcoma, leiomyosarcoma, and osteosarcoma), and a lack of comprehensive clinical data. Gounder et al. present the genetic characteristics of 7494 patients across 44 different sarcoma subtypes. This research sheds light on the potential clinical benefits of utilizing advanced genetic sequencing techniques for diagnosing, prognosis, and managing connective tissue malignancies. For example, the initial diagnoses made by sarcoma pathologists were altered in 4% of patients following the analysis of genomic sequencing results. In these particular cases, two patients initially diagnosed with leiomyosarcoma were reclassified as having dedifferentiated liposarcoma, leading to a change in their treatment approach to include investigational *MDM2* or *CDK4* inhibitors. Additionally, a third patient initially diagnosed with sarcoma NOS was identified as having PEComa due to *TSC2* loss and was recommended treatment with an mTOR inhibitor. Lastly, a fourth patient with MPNST was reclassified as having synovial sarcoma based on detecting an *SS18::SSX2* fusion, leading to an evaluation for NY-ESO-1-based T-cell therapy. In this study, 31.7% of patients had actionable genetic alterations influencing treatment decisions. Actionability definitions varied, highlighting evolving criteria. Genomic profiling informed therapy choices in 29% of patients, but access barriers persisted. The NCI-MATCH study [41] exemplified the gap between genomic research and rare cancer care, emphasizing the need for equity in precision testing and improved clinical trial access.

Molecular profiling holds immense potential for advancing sarcoma patient treatment. Tyrosine kinase inhibitors (TKIs) have become a staple in addressing sarcomas like GIST, where mutations in *KIT* and *PDGFRA* genes drive tumorigenesis. TKIs, such as imatinib, effectively target these mutations, but resistance often emerges due to secondary *KIT* or *PDGFRA* mutations [42]. In other soft tissue sarcomas (STSs), approved targeted therapies are limited to TKIs like pazopanib, which may not effectively target sarcoma stem cells and can lead to resistance. Combining TKIs with inhibitors of other signaling pathways, such as IGF1R/IR or MEK, has been proposed to overcome resistance. Additionally, phosphoproteomic profiling has identified HSP90 inhibition as a potential strategy to overcome resistance [43].

Furthermore, many studies highlight the promise of kinase inhibitors such as larotrectenib in treating *NTRK*-fusion-positive sarcomas [44] and DNA minor groove-binding agents like trabectedin or mithramycin as potential inhibitors of *EWSR1::FLI1*-mediated transcription. While mithramycin faced toxicity challenges, second-generation analogs like EC-8042 offer clinical possibilities [45].

A fascinating pilot study explores the potential of point-of-care nanopore sequencing for methylation-based sarcoma classification, aiming to overcome limitations associated with existing commercial arrays [29]. The customized nanopore pipeline shows promise in diagnosing 11 sarcoma tumor types promptly. However, broader validation across tumor types and centers and statistical refinement are needed. An expanded classifier incorporating multiple data layers is expected to enhance accuracy. This advancement could lead to quicker, point-of-care sarcoma diagnosis and insights into sarcoma biology through methylation patterns, copy-number alteration, and translocation detection.

These findings underscore the importance of comprehensive genomic profiling to identify activated signaling pathways, paving the way for patient-specific treatment regimens and biomarker-guided trials. Understanding interpatient pharmacokinetic variability is also crucial for predicting and addressing resistance. Molecular profiling is poised to usher in a new era of tailored and effective sarcoma treatments. However, given the potential constraints in terms of costs and resources, it is essential to establish a strategic approach for the prudent utilization of NGS and molecular profiling in sarcoma management.

### Artificial intelligence and molecular pathology

The field of diagnostic pathology has become increasingly complex due to advances in both histomorphological and molecular profiling. Pathology has evolved to play a crucial role in diagnosing diseases, estimating prognoses, and predicting precision therapies [46]. This has led to high expectations for applying artificial intelligence (AI) and machine learning, which can analyze intricate data quantitatively and standardizedly, improving diagnostic accuracy. Recent research has shown that predicting specific molecular characteristics is possible based on tissues’ physical appearance or morphology. For example, a recent study from the French Sarcoma Group showcases the potential of deep learning (DL) in predicting the progression risk of localized GIST. While refinement is necessary for clinical application, DL can detect somatic mutations, notably the specific *PDGFRA* exon 18 D842V mutation. This DL method can expedite treatment decisions, particularly for patients with intermediate-risk Miettinen GIST, who typically do not require adjuvant treatment, and high-risk Miettinen GIST, where avapratinib treatment is essential. Furthermore, this approach may prove invaluable in regions with limited access to molecular techniques and serve as a research tool for discovering fresh histological features from whole slide images.

### Enhancing pathologist visibility through involvement in the sarcoma patient experience

The involvement of sarcoma pathologists in the diagnostic process enhances the sarcoma patient experience and sheds light on the pathologist’s vital role [47]. Their expertise is indispensable in the context of precision medicine and shared decision-making. Sarcoma pathologists ensure accurate diagnosis and classification in the correct turnaround time, which is critical for tailoring precise treatments [48]. Pathologists could contribute to a collaborative network by actively engaging with patients, fostering knowledge sharing and synergies. This approach promotes equality in precision medicine. Including patients in advisory boards empowers them in treatment decisions and drives strategies for implementing precision medicine. Interactive meetings facilitate community engagement and promote awareness of the pathologist’s essential contributions to sarcoma care. This holistic approach improves patient outcomes and elevates the visibility and significance of the sarcoma pathologist’s work.

### Ten advice/action points for the next generation of sarcoma pathologists

1. Stay updated on evolving subtypes: Keep learning about emerging sarcoma subtypes and their molecular profiles to ensure accurate diagnosis and classification.
2. Promote data integration: Advocate for seamless integration of molecular data into pathology reports, facilitating informed treatment decisions and enhancing patient care.
3. Foster effective communication: Promote open and effective communication within multidisciplinary teams to ensure a cohesive approach to sarcoma care and research.
4. Advocate for resources: Advocate for adequate resources, including staffing, equipment, and digital pathology infrastructure, to support clinical responsibilities and research commitments.
5. Collaborate actively in research: Actively participate in sarcoma research initiatives, contributing expertise in pathology to advance diagnostic techniques and treatment modalities.
6. Prioritize workload management: Implement strategies for effective workload management, enabling pathologists to balance clinical duties with research involvement.
7. Embrace digital pathology and AI: Embrace digital pathology and artificial intelligence tools, staying updated on their integration into diagnostics and research to enhance accuracy and efficiency.
8. Mentor future pathologists: Dedicate time to mentor and educate the next generation of sarcoma pathologists, ensuring the continuity of expertise in the field.
9. Engage in continuous learning: Commit to ongoing learning and professional development to remain at the forefront of sarcoma pathology advancements.
10. Advocate for patient-centered care: Champion a patient-centered approach within multidisciplinary teams, ensuring patients’ unique needs and perspectives are considered in research and care decisions.

By addressing these action points, sarcoma pathologists can overcome the challenges they face and continue to play a pivotal role in advancing research and enhancing the care of sarcoma patients.

## Conclusion

Molecular pathologists in bone and soft tissue sarcomas (BSTPath) face various challenges. The pre-analytical phase is intricate due to the need for decalcification in bone sarcomas, impacting nucleic acid quality. Biopsy collection is complicated because of deep-seated tumors and diverse subtypes, emphasizing RNA integrity preservation. Ensuring equitable access to advanced diagnostics for the 50 + sarcoma subtypes is crucial, emphasizing the role of healthcare systems in availability. Managing sarcoma samples effectively, especially when repeated biopsies are not possible, is vital, with multigene tests preserving original diagnostic biopsy blocks. Liquid biopsies and analyzing circulating biomarkers offer promise but require standardization and validation due to sarcoma rarity and heterogeneity. Immunotherapy’s evolving role in sarcomas necessitates ongoing biomarker validation by molecular pathologists. As targeted therapies emerge, pathologists detect resistance mechanisms, enabling personalized treatment plans. Multidisciplinary tumor boards are essential for sarcoma care, with molecular insights guiding treatment decisions. National NGS networks streamline diagnostics, exemplified by French and Spanish initiatives. Molecular pathology advances through comprehensive genome profiling, kinase inhibitors, and innovative diagnostic techniques like nanopore sequencing. Artificial intelligence aids histomorphological and molecular analysis, improving accuracy. Involving sarcoma pathologists in patient care enhances the patient experience and their visibility. The focus of future sarcoma molecular pathologists will include staying updated, promoting data integration, fostering communication, advocating for resources, active research involvement, workload management, embracing digital pathology and AI, mentoring, continuous learning, and supporting patient-centered care. These efforts address BSTPath challenges, shaping the future of sarcoma care.

## Data Availability

The data and original images that support the findings of this study are available on request from the corresponding author, [EDA].

## References

[1] WHO Classification of Tumours Editorial Board (2020) Soft tissue and bone tumours. WHO classification of tumours. Vol. 3. 5th ed. Lyon: IARC Publications

[2] Kallen ME, Hornick JL (2021). The 2020 WHO classification: what’s new in soft tissue tumor pathology?. Am J Surg Pathol.

[3] Sbaraglia M, Bellan E, Dei Tos AP (2021). The 2020 WHO classification of soft tissue tumours: news and perspectives. Pathologica.

[4] Setty BA, Jinesh GG, Arnold M, Pettersson F, Cheng CH, Cen L, Yoder SJ, Teer JK, Flores ER, Reed DR, Brohl AS (2020). The genomic landscape of undifferentiated embryonal sarcoma of the liver is typified by C19MC structural rearrangement and overexpression combined with TP53 mutation or loss. PLoS Genet.

[5] Spunt SL, Million L, Chi YY, Anderson J, Tian J, Hibbitts E, Coffin C, McCarville MB, Randall RL, Parham DM, Black JO, Kao SC, Hayes-Jordan A, Wolden S, Laurie F, Speights R, Kawashima E, Skapek SX, Meyer W, Pappo AS, Hawkins DS (2020). A risk-based treatment strategy for non-rhabdomyosarcoma soft-tissue sarcomas in patients younger than 30 years (ARST0332): a Children’s Oncology Group prospective study. Lancet Oncol.

[6] Lam SW, Silva TM, Bovée JVMG (2022). New molecular entities of soft tissue and bone tumors. Curr Opin Oncol.

[7] Oda Y, Yamamoto H, Kohashi K, Yamada Y, Iura K, Ishii T, Maekawa A, Bekki H (2017). Soft tissue sarcomas: from a morphological to a molecular biological approach. Pathol Int.

[8] Papke DJ, Hornick JL (2022). Recent advances in the diagnosis, classification and molecular pathogenesis of cutaneous mesenchymal neoplasms. Histopathology.

[9] Stacchiotti S, Miah AB, Frezza AM, Messiou C, Morosi C, Caraceni A, Antonescu CR, Bajpai J, Baldini E, Bauer S, Biagini R, Bielack S, Blay JY, Bonvalot S, Boukovinas I, Bovee JVMG, Boye K, Brodowicz T, Callegaro D, De Alava E, Deoras-Sutliff M, Dufresne A, Eriksson M, Errani C, Fedenko A, Ferraresi V, Ferrari A, Fletcher CDM, Garcia Del Muro X, Gelderblom H, Gladdy RA, Gouin F, Grignani G, Gutkovich J, Haas R, Hindi N, Hohenberger P, Huang P, Joensuu H, Jones RL, Jungels C, Kasper B, Kawai A, Le Cesne A, Le Grange F, Leithner A, Leonard H, Lopez Pousa A, Martin Broto J, Merimsky O, Merriam P, Miceli R, Mir O, Molinari M, Montemurro M, Oldani G, Palmerini E, Pantaleo MA, Patel S, Piperno-Neumann S, Raut CP, Ravi V, Razak ARA, Reichardt P, Rubin BP, Rutkowski P, Safwat AA, Sangalli C, Sapisochin G, Sbaraglia M, Scheipl S, Schöffski P, Strauss D, Strauss SJ, Sundby Hall K, Tap WD, Trama A, Tweddle A, van der Graaf WTA, Van De Sande MAJ, Van Houdt W, van Oortmerssen G, Wagner AJ, Wartenberg M, Wood J, Zaffaroni N, Zimmermann C, Casali PG, Dei Tos AP, Gronchi A (2021). Epithelioid hemangioendothelioma, an ultra-rare cancer: a consensus paper from the community of experts. ESMO Open.

[10] Whaley RD, Thompson LDR (2021). Epstein-Barr virus-associated smooth muscle tumors of larynx: a clinicopathologic study and comprehensive literature review of 12 cases. Head Neck Pathol.

[11] Kao YC, Bennett JA, Suurmeijer AJH, Dickson BC, Swanson D, Wanjari P, Zhang L, Lee JC, Antonescu CR (2021). Recurrent MEIS1-NCOA2/1 fusions in a subset of low-grade spindle cell sarcomas frequently involving the genitourinary and gynecologic tracts. Mod Pathol.

[12] Liu YJ, Wang W, Yeh J, Wu Y, Mantilla JG, Fletcher CDM, Ricciotti RW, Chen EY (2021). Calcified chondroid mesenchymal neoplasms with FN1-receptor tyrosine kinase gene fusions including FGFR2, FGFR1, MERTK, NTRK1, and TEK: a molecular and clinicopathologic analysis. Mod Pathol.

[13] Wang L, Zehir A, Sadowska J, Zhou N, Rosenblum M, Busam K, Agaram N, Travis W, Arcila M, Dogan S, Berger MF, Cheng DT, Ladanyi M, Nafa K, Hameed M (2015). Consistent copy number changes and recurrent PRKAR1A mutations distinguish melanotic schwannomas from melanomas: SNP-array and next generation sequencing analysis. Genes Chromosomes Cancer.

[14] Cidre-Aranaz F, Watson S, Amatruda JF, Nakamura T, Delattre O, de Alava E, Dirksen U, Grünewald TGP (2022). Small round cell sarcomas. Nat Rev Dis Primers.

[15] Chen CH, Chang KC, Chuang CH, Fu JT, Huang HY (2022). The emerging PRRX1-NCOA fibroblastic neoplasm: a combined reappraisal of published tumors and two new cases. Virchows Arch.

[16] Mancini I, Righi A, Gambarotti M, Picci P, Dei Tos AP, Billings SD, Simi L, Franchi A (2017). Phenotypic and molecular differences between giant-cell tumour of soft tissue and its bone counterpart. Histopathology.

[17] Odate T, Satomi K, Kubo T, Matsushita Y, Ueno T, Kurose A, Shomori K, Nakai T, Watanabe R, Segawa K, Ohshika S, Miyake N, Kudo S, Shimoi T, Kobayashi E, Komiyama M, Yoshimoto S, Nakatani F, Kawai A, Yatabe Y, Kohsaka S, Ichimura K, Ichikawa H, Yoshida A (2023). Inflammatory rhabdomyoblastic tumor: clinicopathologic and molecular analysis of 13 cases. Mod Pathol.

[18] Cordier F, Van der Meulen J, Van Gaever B, Lapeire L, Sys G, Van Dorpe J, Creytens D (2022). Undifferentiated sarcoma of bone with a round to epithelioid cell phenotype harboring a novel EWSR1-SSX2 fusion identified by RNA-based next-generation sequencing. Genes Chromosomes Cancer.

[19] Parrack PH, Mariño-Enríquez A, Fletcher CDM, Hornick JL, Papke DJ (2023). GLI1 immunohistochemistry distinguishes mesenchymal neoplasms with GLI1 alterations from morphologic mimics. Am J Surg Pathol.

[20] Baumhoer D, Amary F, Flanagan AM (2019). An update of molecular pathology of bone tumors. Lessons learned from investigating samples by next generation sequencing. Genes Chromosomes Cancer.

[21] Cordier F, Creytens D (2023). New kids on the block: FOS and FOSB gene. J Clin Pathol.

[22] Bovée JV, Hogendoorn PC (2019). Non-ossifying fibroma: a RAS-MAPK driven benign bone neoplasm. J Pathol.

[23] Wen X, Cimera R, Aryeequaye R, Abhinta M, Athanasian E, Healey J, Fabbri N, Boland P, Zhang Y, Hameed M (2021). Recurrent loss of chromosome 22 and SMARCB1 deletion in extra-axial chordoma: a clinicopathological and molecular analysis. Genes Chromosomes Cancer.

[24] Italiano A, Di Mauro I, Rapp J, Pierron G, Auger N, Alberti L, Chibon F, Escande F, Voegeli AC, Ghnassia JP, Keslair F, Laé M, Ranchère-Vince D, Terrier P, Baffert S, Coindre JM, Pedeutour F (2016). Clinical effect of molecular methods in sarcoma diagnosis (GENSARC): a prospective, multicentre, observational study. Lancet Oncol.

[25] Bovée JV, Hogendoorn PC (2010). Molecular pathology of sarcomas: concepts and clinical implications. Virchows Arch.

[26] Vyse S, Thway K, Huang PH, Jones RL (2021). Next-generation sequencing for the management of sarcomas with no known driver mutations. Curr Opin Oncol.

[27] Chang KTE, Goytain A, Tucker T, Karsan A, Lee CH, Nielsen TO, Ng TL (2018). Development and evaluation of a pan-sarcoma fusion gene detection assay using the NanoString nCounter platform. J Mol Diagn.

[28] Koelsche C, Schrimpf D, Stichel D, Sill M, Sahm F, Reuss DE, Blattner M, Worst B, Heilig CE, Beck K, Horak P, Kreutzfeldt S, Paff E, Stark S, Johann P, Selt F, Ecker J, Sturm D, Pajtler KW, Reinhardt A, Wefers AK, Sievers P, Ebrahimi A, Suwala A, Fernández-Klett F, Casalini B, Korshunov A, Hovestadt V, Kommoss FKF, Kriegsmann M, Schick M, Bewerunge-Hudler M, Milde T, Witt O, Kulozik AE, Kool M, Romero-Pérez L, Grünewald TGP, Kirchner T, Wick W, Platten M, Unterberg A, Uhl M, Abdollahi A, Debus J, Lehner B, Thomas C, Hasselblatt M, Paulus W, Hartmann C, Staszewski O, Prinz M, Hench J, Frank S, Versleijen-Jonkers YMH, Weidema ME, Mentzel T, Griewank K, de Álava E, Martín JD, Gastearena MAI, Chang KT, Low SYY, Cuevas-Bourdier A, Mittelbronn M, Mynarek M, Rutkowski S, Schüller U, Mautner VF, Schittenhelm J, Serrano J, Snuderl M, Büttner R, Klingebiel T, Buslei R, Gessler M, Wesseling P, Dinjens WNM, Brandner S, Jaunmuktane Z, Lyskjær I, Schirmacher P, Stenzinger A, Brors B, Glimm H, Heining C, Tirado OM, Sáinz-Jaspeado M, Mora J, Alonso J, Del Muro XG, Moran S, Esteller M, Benhamida JK, Ladanyi M, Wardelmann E, Antonescu C, Flanagan A, Dirksen U, Hohenberger P, Baumhoer D, Hartmann W, Vokuhl C, Flucke U, Petersen I, Mechtersheimer G, Capper D, Jones DTW, Fröhling S, Pfister SM, von Deimling A (2021). Sarcoma classification by DNA methylation profiling. Nat Commun.

[29] Iluz A, Maoz M, Lavi N, Charbit H, Or O, Olshinka N, Demma JA, Adileh M, Wygoda M, Blumenfeld P, Gliner-Ron M, Azraq Y, Moss J, Peretz T, Eden A, Zick A, Lavon I (2023). Rapid classification of sarcomas using methylation fingerprint: a pilot study. Cancers (Basel).

[30] Bovée JVMG, Webster F, Amary F, Baumhoer D, Bloem JLH, Bridge JA, Cates JMM, de Alava E, Dei Tos AP, Jones KB, Mahar A, Nielsen GP, Righi A, Wagner AJ, Yoshida A, Fletcher CDM (2023). Datasets for the reporting of primary tumour in bone: recommendations from the International Collaboration on Cancer Reporting (ICCR). Histopathology.

[31] https://www.iccr-cancer.org/datasets/published-datasets/soft-tissue-bone/ accessed November 1, 2023

[32] Marcilla D, Machado I, Grünewald TGP, Llombart-Bosch A, de Álava E (2021). (Immuno)histological analysis of Ewing sarcoma. Methods Mol Biol.

[33] Blanchi J, Taleb S, Bayle A, Verret B, Toulmonde M, Spalato-Ceruso M, Dubos P, Laizet Y, Alame M, Khalifa E, Italiano A (2023). Clinical utility of circulating tumor DNA sequencing with a large panel in patients with advanced soft-tissue sarcomas. Cancer Commun (Lond).

[34] Grünewald TG, Alonso M, Avnet S, Banito A, Burdach S, Cidre-Aranaz F, Di Pompo G, Distel M, Dorado-Garcia H, Garcia-Castro J, González-González L, Grigoriadis AE, Kasan M, Koelsche C, Krumbholz M, Lecanda F, Lemma S, Longo DL, Madrigal-Esquivel C, Morales-Molina Á, Musa J, Ohmura S, Ory B, Pereira-Silva M, Perut F, Rodriguez R, Seeling C, Al Shaaili N, Shaabani S, Shiavone K, Sinha S, Tomazou EM, Trautmann M, Vela M, Versleijen-Jonkers YM, Visgauss J, Zalacain M, Schober SJ, Lissat A, English WR, Baldini N, Heymann D (2020). Sarcoma treatment in the era of molecular medicine. EMBO Mol Med.

[35] Wilding CP, Elms ML, Judson I, Tan AC, Jones RL, Huang PH (2019). The landscape of tyrosine kinase inhibitors in sarcomas: looking beyond pazopanib. Expert Rev Anticancer Ther.

[36] Péron J, Marreaud S, Staelens D, Raveloarivahy T, Nzokirantevye A, Flament J, Steuve J, Lia M, Collette L, Schöffski P (2019). A multinational, multi-tumour basket study in very rare cancer types: the European Organization for Research and Treatment of Cancer phase II 90101 ‘CREATE’ trial. Eur J Cancer.

[37] Cardoso E, Guidi M, Blanchet B, Schneider MP, Decosterd LA, Buclin T, Csajka C, Widmer N (2020). Therapeutic drug monitoring of targeted anticancer protein kinase inhibitors in routine clinical use: a critical review. Ther Drug Monit.

[38] Berclaz LM, Burkhard-Meier A, Lange P, Di Gioia D, Schmidt M, Knösel T, Klauschen F, von Bergwelt-Baildon M, Heinemann V, Greif PA, Westphalen CB, Heinrich K, Lindner LH (2023). Implementing precision oncology for sarcoma patients: the CCCLMUmolecular tumor board experience. J Cancer Res Clin Oncol.

[39] https://www.proyectoimperas.com/ accessed Nov 1, 2023

[40] Gounder MM, Agaram NP, Trabucco SE, Robinson V, Ferraro RA, Millis SZ, Krishnan A, Lee J, Attia S, Abida W, Drilon A, Chi P, Angelo SP, Dickson MA, Keohan ML, Kelly CM, Agulnik M, Chawla SP, Choy E, Chugh R, Meyer CF, Myer PA, Moore JL, Okimoto RA, Pollock RE, Ravi V, Singh AS, Somaiah N, Wagner AJ, Healey JH, Frampton GM, Venstrom JM, Ross JS, Ladanyi M, Singer S, Brennan MF, Schwartz GK, Lazar AJ, Thomas DM, Maki RG, Tap WD, Ali SM, Jin DX (2022). Clinical genomic profiling in the management of patients with soft tissue and bone sarcoma. Nat Commun.

[41] Flaherty KT, Gray R, Chen A, Li S, Patton D, Hamilton SR, Williams PM, Mitchell EP, Iafrate AJ, Sklar J, Harris LN, McShane LM, Rubinstein LV, Sims DJ, Routbort M, Coffey B, Fu T, Zwiebel JA, Little RF, Marinucci D, Catalano R, Magnan R, Kibbe W, Weil C, Tricoli JV, Alexander B, Kumar S, Schwartz GK, Meric-Bernstam F, Lih CJ, McCaskill-Stevens W, Caimi P, Takebe N, Datta V, Arteaga CL, Abrams JS, Comis R, O’Dwyer PJ, Conley BA, NCI-MATCH Team (2020). The Molecular Analysis for Therapy Choice (NCI-MATCH) trial: lessons for genomic trial design. J Natl Cancer Inst.

[42] Casali PG, Abecassis N, Aro HT, Bauer S, Biagini R, Bielack S, Bonvalot S, Boukovinas I, Bovee JVMG, Brodowicz T (2018). Gastrointestinal stromal tumours: ESMO-EURACAN clinical practice guidelines for diagnosis, treatment and follow-up. Ann Oncol.

[43] Lanzi C, Dal Bo L, Favini E, Tortoreto M, Beretta GL, Arrighetti N, Zaffaroni N, Cassinelli G (2019). Overactive IGF1/insulin receptors and NRASQ61R mutation drive mechanisms of resistance to pazopanib and define rational combination strategies to treat synovial sarcoma. Cancers (Basel).

[44] Demetri GD, Antonescu CR, Bjerkehagen B, Bovée JVMG, Boye K, Chacón M, Dei Tos AP, Desai J, Fletcher JA, Gelderblom H, George S, Gronchi A, Haas RL, Hindi N, Hohenberger P, Joensuu H, Jones RL, Judson I, Kang YK, Kawai A, Lazar AJ, Le Cesne A, Maestro R, Maki RG, Martín J, Patel S, Penault-Llorca F, Premanand Raut C, Rutkowski P, Safwat A, Sbaraglia M, Schaefer IM, Shen L, Serrano C, Schöffski P, Stacchiotti S, Sundby Hall K, Tap WD, Thomas DM, Trent J, Valverde C, van der Graaf WTA, von Mehren M, Wagner A, Wardelmann E, Naito Y, Zalcberg J, Blay JY (2020). Diagnosis and management of tropomyosin receptor kinase (TRK) fusion sarcomas: expert recommendations from the World Sarcoma Network. Ann Oncol.

[45] Estupiñán Ó, Rey V, Tornín J, Murillo D, Gallego B, Huergo C, Blanco-Lorenzo V, Victoria González M, Rodríguez A, Moris F, González J, Ayllón V, Ramos-Mejía V, Bigas A, Rodríguez R (2023). Abrogation of stemness in osteosarcoma by the mithramycin analog EC-8042 is mediated by its ability to inhibit NOTCH-1 signaling. Biomed Pharmacother.

[46] Stenzinger A, Alber M, Allgäuer M, Jurmeister P, Bockmayr M, Budczies J, Lennerz J, Eschrich J, Kazdal D, Schirmacher P, Wagner AH, Tacke F, Capper D, Müller KR, Klauschen F (2022). Artificial intelligence and pathology: from principles to practice and future applications in histomorphology and molecular profiling. Semin Cancer Biol.

[47] https://thepathologist.com/outside-the-lab/the-invisible-doctor accessed Nov 1, 2023

[48] Martin S, Clark SE, Gerrand C, Gilchrist K, Lawal M, Maio L, Martins A, Storey L, Taylor RM, Wells M, Whelan JS, Windsor R, Woodford J, Vindrola-Padros C, Fern LA (2023). Patients’ experiences of a sarcoma diagnosis: a process mapping exercise of diagnostic pathways. Cancers (Basel).

